# Reverse Engineering Boolean Networks: From Bernoulli Mixture Models to Rule Based Systems

**DOI:** 10.1371/journal.pone.0051006

**Published:** 2012-12-17

**Authors:** Mehreen Saeed, Maliha Ijaz, Kashif Javed, Haroon Atique Babri

**Affiliations:** 1 Department of Computer Science/FAST, National University of Computer and Emerging Sciences, Lahore, Pakistan; 2 FAST, National University of Computer and Emerging Sciences, Lahore, Pakistan; 3 Department of Electrical Engineering/University of Engineering and Technology, Lahore, Pakistan; University of Glasgow, United Kingdom

## Abstract

A Boolean network is a graphical model for representing and analyzing the behavior of gene regulatory networks (GRN). In this context, the accurate and efficient reconstruction of a Boolean network is essential for understanding the gene regulation mechanism and the complex relations that exist therein. In this paper we introduce an elegant and efficient algorithm for the reverse engineering of Boolean networks from a time series of multivariate binary data corresponding to gene expression data. We call our method ReBMM, i.e., reverse engineering based on Bernoulli mixture models. The time complexity of most of the existing reverse engineering techniques is quite high and depends upon the indegree of a node in the network. Due to the high complexity of these methods, they can only be applied to sparsely connected networks of small sizes. ReBMM has a time complexity factor, which is independent of the indegree of a node and is quadratic in the number of nodes in the network, a big improvement over other techniques and yet there is little or no compromise in accuracy. We have tested ReBMM on a number of artificial datasets along with simulated data derived from a plant signaling network. We also used this method to reconstruct a network from real experimental observations of microarray data of the yeast cell cycle. Our method provides a natural framework for generating rules from a probabilistic model. It is simple, intuitive and illustrates excellent empirical results.

## Introduction

Boolean networks were introduced by Kauffman in the sixties and were one of the first methods to describe gene expression data [1] and model a gene regulatory network (GRN). GRNs have been the focus of research in the bioinformatics and genome sciences community for more than a decade now. A GRN can be viewed as a network composed of nodes and edges, where nodes represent genes or proteins and edges symbolize various relationships, at the molecular level, such as protein-protein or DNA-protein interactions [2]. There are many existing techniques for modeling GRNs, e.g., Bayesian networks [3], neural networks [4], support vector machines [5], metabolic control analysis [6] etc. A review of inference techniques of GRNs has been presented by Hecker *et al.* [2]. The problems and methods related to GRNs have also been outlined by Han *et al.* [7], where they discuss the various network topologies and the reconstruction methods. One technique used to model GRNs is based on Boolean network models.

Boolean networks are constructed by discretizing the expression data to two states, zero indicating ‘off’ state and one indicating ‘on’. The state of any node at any time is determined by its parent nodes and the updating scheme, i.e., synchronous or asynchronous. This is a very strong simplification with respect to real GRNs that may not be binary, but it has been shown that “meaningful biological information” is still present in data even if it is quantized to two possible states [8], [9]. There are also effective methods to discretized gene expression data to binary form (see e.g., [10]).

In this article we present a fast, efficient and accurate method called ReBMM (reverse engineering of Boolean networks using Bernoulli mixture models) for reverse engineering Boolean Networks by making use of a probabilistic model called a Bernoulli mixture model. We illustrate the reconstruction of a Boolean network from state transition data that represents a time series of gene expression data. Our method is based on learning Bernoulli mixture models from raw data and these mixtures are then used to determine the network structure and also the logical rules governing each node in the network. Most of the existing reverse engineering algorithms have a high time complexity, normally a polynomial of a high degree or an exponential in some cases [11]–[15]. Their application is therefore restricted to small networks. In this work we show that ReBMM has significantly reduced time complexity as compared to other methods and yet it predicts a Boolean network with little or no compromise in accuracy.

When reverse engineering Boolean networks, two problems have to be addressed. One is to determine the network structure in which the parents of each individual node have to be determined. Secondly, the rules governing each node in the network have to be inferred from the given data. A majority of the state of the art reverse engineering algorithms generally involve a combinatorial search through the space of all node combinations to reconstruct a Boolean network, leading to a high time complexity. Normally these techniques limit the indegree, 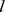, of a node to a small number like 3 or 4. Some examples of reconstruction algorithms include REVEAL, which is based on the information theoretic principle and has a time complexity factor a multiple of 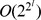 [11]; predictor chooser method of Ideker *et al.* that uses minimum set covering [13]; minimal sets algorithm and genetic algorithms by Dimitrova *et al.* [16] Monte-Carlo type randomized algorithm of Akutsu *et al.* [12], which has an exponential time complexity. Best fit extension principle of Lähdesmäki *et al.* again involves a factor of 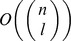 [14], where 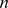 is the total number of nodes in the network. Nam *et al.* devised an efficient search algorithm for learning Boolean networks [15], with a complexity of 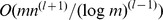, where 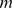 is the total number of time steps (explained later). For all the afore mentioned methods, the performance deteriorates considerably with an increase in the indegree and the size of the network.

Recently Maucher *et al.* introduced a very elegant algorithm for reconstructing Boolean networks by using correlations. The method has a time complexity quadratic in the number of nodes in the network [17]. Their approach is one of the first methods which is independent of the indegree of a node. However, their algorithm is only restricted to carrying out step 1 of the algorithm, i.e., the structure of the network is determined but the set of rules governing each node in the network is not determined. Their method is also restricted to reverse engineering networks, which have only Boolean monotone functions like the logical AND and the logical OR and cannot deal with functions like the XOR.

In this paper, we introduce ReBMM, a method that infers a Boolean network and has a complexity quadratic in terms of the number of nodes in the network and does not depend upon the indegree of a node. The method is simple and intuitive and based on a probabilistic model, which is later converted to a rule based system using a very simple conversion technique. We show how Bernoulli mixture models can be used to determine the parents of a node and how these mixtures lend themselves to a natural representation of logical Boolean rules, an approach which has not been explored before. Our method can also infer both monotone and non-monotone functions.

ReBMM is an intuitive method that derives a rule based system from a probabilistic model based on Bernoulli mixture models. Finite mixture models of probability distributions represent a convex combination of different parametric distributions, where each distribution has its own set of parameters [18], [19]. These mixtures are used in situations where the actual shape of the distribution is unknown, and a single distribution is not sufficient to model the local variations in data. Previously, finite mixture models have been widely used in cluster analysis, where the task is to find entities of a similar nature and to group them together [20]. In this paper we extend the usage of mixture models to extract rules and summaries from data.

ReBMM was devised as a model to explain the data derived from a plant signaling network called the SIGNET dataset (see http://www.causality.inf.ethz.ch/pot-luck.php), [21], [22]. This was launched as a part of the causality challenge, which consisted of different tasks related to causal discovery [23]. We developed this method to analyze and infer asynchronous Boolean networks. In this paper we extend our work to synchronous networks and also carry out a detailed analysis of the complexity of this method. We have also devised a model selection scheme for this method and tested it on synthetic and real life datasets. Finally we demonstrate how this algorithm can be applied to bigger networks with varying indegrees of individual nodes.

## Methods

Boolean networks were developed by Stuart Kauffman in 1969, as a model for the genetic regulatory network and as a network of logical elements [1]. He showed that many biological systems can be modeled by sparsely connected networks. A Boolean network can be represented as a directed graph, where nodes can be the genes or the external factors affecting the genes or even the states of different proteins [24].

**Figure 1:**
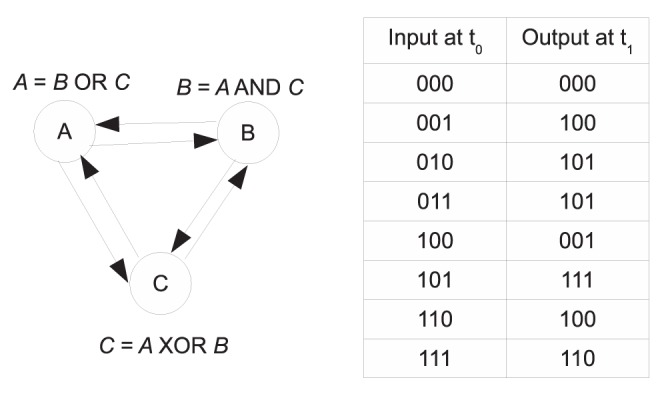
An example Boolean network and associated truth table.

We picture a Boolean network as a directed graph 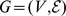, with 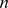 nodes/vertices and the value at each vertex/node is denoted by 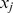. Each node can be in one of the two possible states, i.e., ‘
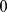
’ or ‘
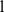
’. Every node is associated with a logical function given by 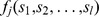, where 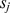 is one of the parents of node 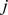 and 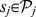, where 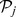, is the set, of all parents of node 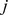. The parent nodes serve as input to the node 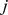. 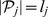 is also called the indegree of node 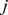. The state of the node at any time step can be determined by the state of the input nodes 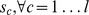, and the Boolean function governing the behavior of the node. It is assumed that the Boolean function 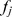 and the set of parents 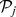 of node 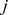 do not change with time. A node may have no connection (0 indegree) or at the most 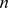 connections (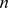 indegree). If a node is connected to itself then we have a self loop. For our current work, we do not consider self loops, however our current framework can be easily extended to incorporate them. Also, in this paper we focus on Boolean networks with a synchronous updating scheme. Here, all the nodes are updated at once, and the state, 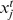, of node 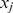 at time 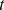, is completely determined by the state of its parent nodes at time 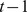, i.e.,
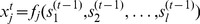
(1)It is also assumed that different nodes can have different indegrees. An example of a Boolean network, and its associated truth table is shown in Figure 1. Here the state of each node at time step 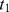 is completely determined by its state at time step 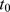. Our goal is to reverse engineer a Boolean network, given a sequence or time series of observations.

### Bernoulli Mixture Models

For real life problems, it is often the case, that a single distribution is not enough to represent an entire dataset. In such instances, a multi-modal distribution might be required and a non-parametric density estimation method can be used to represent the dataset. An alternate solution is to use a mixture of distributions. In this case, we assume that there are multiple distribution sources, which are generating the data. Each distribution has its own set of parameters [19], [25]–[27]. In the text that follows, we assume that we have binary input data and we'll use a distribution based on Bernoulli mixtures to model it.

A Bernoulli mixture model is an extension of a simple Bernoulli distribution. A Bernoulli distribution is used to model binary data, where a random variable can have only two possible values, i.e., true or false. Suppose we have a dataset consisting of 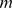 vectors, 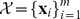, where 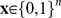. In the context of Boolean networks, 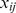 represents the state of node 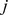 in the 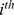 data point. A single univariate Bernoulli distribution has only one parameter, i.e., the probability 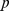 that the value of the random variable is true. The probability that the value of the variable is false is automatically derived as 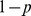. The maximum likelihood estimate of the sufficient statistic, 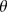 is 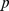, which is estimated using the mean value of the samples of 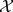. In case of multivariate data, the distribution assumes that each feature is independent, and hence the form of this distribution for a single 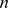-dimensional random variable 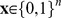, given the parameters of the distribution is expressed as [19]:
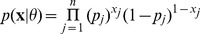
(2)The sufficient statistic for this distribution is the probability vector 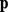, which represents the probability of each feature assuming the value true. 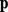 also represents the mean vector of the distribution, i.e.,
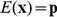
(3)We can extend the concept of a multivariate Bernoulli distribution to a mixture model composed of 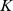 Bernoulli distributions, each mixture or component having its own set of parameters, i.e., 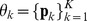. Here, the probability of a single data point is given by:
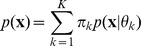
where 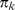 is the prior of the 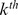 mixture. In this case we require 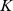 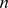-dimensional probability vectors and 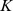 priors to fully describe a Bernoulli mixture model, hence, the parameters involved in this model are 
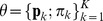
.

Interestingly, a single Bernoulli distribution assumes feature independence, however, with multiple Bernoulli distributions, we can capture the relationships that exist between the different features. The covariance matrix of this distribution is no longer a diagonal matrix, which enables us to analyze the correlations between individual attributes. The mean of this distribution is given by [19]:
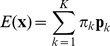
(4)and the covariance matrix is given by [19]:
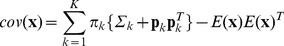
(5)where 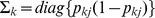. The correlations between variables can thus be computed from the covariance matrix.

### Learning a Bernoulli mixture model from data

The Bernoulli mixtures representing the data are not unique and the problem of identifying Bernoulli mixture model parameters is intractable [28]. However, we can use some optimization criterion to iteratively estimate the mixture parameters. Here, we will describe the well known optimization technique called expectation maximization EM, introduced by Dempster *et al.* [18] to learn the components of a mixture model. The objective function maximized by EM is the log likelihood function given by:
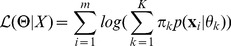
(6)where 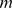 is the total number of data points. To optimize the above objective function, EM associates a vector of hidden/latent variables 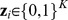 with each data point. 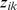 is an indicator variable with 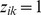 if the 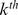 mixture component generated the 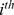 example point and zero otherwise.

EM estimates the mixture parameters, iteratively, in two steps. The first step is called the expectation step or E-step, which estimates the expected value of the latent variables, keeping the parameters, 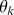, of each distribution, fixed. The second step is known as the maximization step or M-step, which re-estimates the parameters 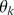, assuming that the values of the hidden variable, 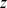, are fixed.

The E-step for a Bernoulli mixture model is given by:
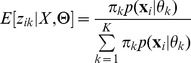
(7)

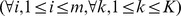
. Here, the expression for 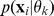 is given by (2).

The M-step updates the model parameters 
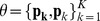
 as:
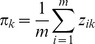
(8)

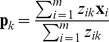
(9)We can use Laplacian priors to get smooth estimates for the probabilities:
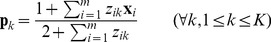
(10)We use the regularized version of M-Step given by [29]:
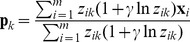
(11)where 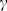 is the regularization constant. To start the EM algorithm we initialize the probabilities with random values and input a large 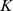 value so that some mixtures/components are automatically annihilated. Also, we use the Laplacian prior to smooth the probability estimates, hence, the probability values are calculated as below:
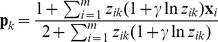
(12)Although EM is a heuristic based method but due to this regularization term for a particular value of 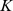, we generally end up with the same parameters of the Bernoulli mixture models even if different initial values of probabilities are used. In addition to using the regularized version of EM algorithm, we also used the training set accuracy as a criterion for selecting the value of the parameter 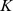. This criterion is discussed later.

### Extracting rules from data using a Bernoulli mixture model

We now describe a general framework for converting a probabilistic system based on Bernoulli mixtures into a rule based system. We use this framework to extract Boolean rules from Bernoulli mixtures, which model raw data. Such a method helps us understand the inter-relations, which exist between the individual nodes of a Boolean network. It is also a simple method for detecting causal elements or parents of a given node in the network. The main steps of our algorithm are outlined in Algorithm 1.

**Table:** 

**Algorithm 1:** The ReBMM Algorithm

1. For each node/variable 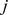 in the dataset repeat the following:
(a) Preprocess raw input data: Build the training data by using variable 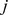 as the target/class and the values of the rest of variables as input data. Leave only one point attractor and omit the rest of repeated point attractors
(b) Repeat for different values of 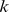 , the total number of mixtures
i. Partition the data into two matrices. One associated with 0 output value and the other associated with 1 output value
ii. Generate 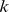 mixtures 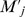 and 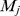 for the corresponding 0 and 1 output values
iii. Generate the set of main vectors 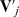 and 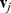 from both 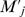 and 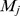 , respectively
iv. Select the parent nodes from the main vectors
v. Simplify a rule
vi. Generate two Boolean rules from 0 and 1 class labels
vii. Check the training accuracy for both classes and output the rule with maximum training accuracy
(c) Select the rule, which has maximum training accuracy, out of all the rules generated from different values of 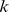

#### Preprocessing Raw Input Data

The raw data available to us for generating a Boolean network comes in the form of a time series of 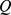 simulations, each simulation consisting of a 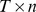 matrix of binary values. Each row represents the state of all nodes of the network at time step 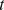. We pre-process the data, by considering each node as a target/output node and associating with it a corresponding input matrix from the rest of the nodes. Each row of the matrix corresponds to an input (at time step 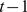) to the value of the target node at time 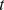. The maximum order of the input matrix is thus, 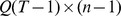, however, we reduce the size of this input matrix. As all simulations result in stable values, we remove duplicate attractor points from our data. Also, it should be noted that we used all stable and unstable values to generate the Boolean rules from data. Our algorithm is limited to removing point attractors and does not detect cycle attractors.

Once we pre-process the raw input data and extract the input matrix, we have an input matrix associated with the output values of a node 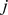. Node 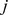 has two possible output/target values, i.e., 0 or 1. Each output has an input vector associated with it. We partition the input rows into two parts, one set of rows associated with output 0 and one set of rows associated with output 1.

#### Concept of Main Vectors and Rule Generation From Bernoulli Mixtures

After pre-processing data for node 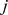, we have input data points associated with 0 and 1 output values of node 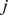. We generate Bernoulli mixtures from each of the two sets of input vectors separately, so that we end up with mixtures 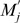 and 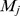 for the corresponding 0 and 1 output values. Now we illustrate how these mixtures can be used for generating Boolean rules. Consider a single mixture/cluster, defined using the probability vector 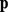. Let us define the notion of a main vector 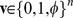, extracted from a probability vector 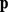. Here, 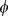 is a don't care value. The values of probability higher than a certain threshold 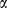 are set to binary true and the values below 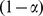 are set to false. Any other value around 0.5 is set to a don't care value. The components of main vector are given by:
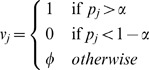
(13)If we make 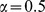, (and convert the 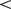 to 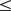) then we don't have any don't care values in the main vector and 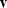 is purely a binary vector, 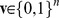. The main vectors represent areas of high data density on the corners of an 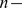
cube. A feature is a literal and an individual corner can be viewed as a conjunction of literals. Together the set, of corners, represents a set of logical rules connected together by the logical ‘OR’ relation, hence, giving us the disjunctive normal form of a rule showing the interrelationships between different features. Hence, we can transform the Bernoulli mixture model into main vectors, from which we can generate a logical rule. From one mixture we can derive a rule involving conjuncts of literals and the total number of mixtures determines the total terms in the disjunctive normal form.

**Figure 2:**
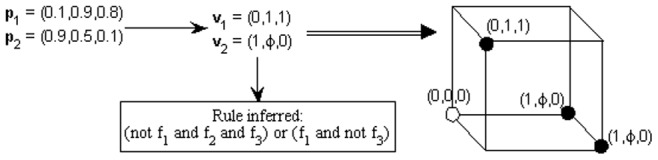
Rule generation: an example with two mixtures [32]. 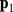
 and 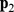 are probability vectors thresholded to main vectors 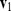 and 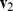, which in turn are mapped onto the corners of a hypercube. A rule is automatically derived from the main vectors and is shown in the box.

Figure 2 illustrates an example of rule mining using the notion of main vectors in 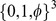. There are two mixtures/clusters and hence two corresponding main vectors, which represent the corners of high data density of a 3-cube. The corresponding Boolean rules are also shown in the figure. Features having a don't care value don't form part of a rule and it can be seen from the figure that a don't care value enables us to represent multiple corners of the hypercube simultaneously, for example, 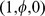 represents both the corners 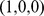 and 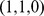


#### Selecting the Parent Nodes

**Figure 3:**
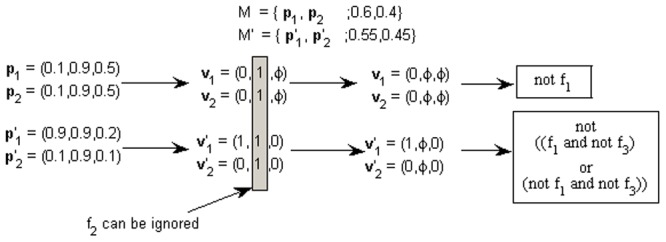
Generation of rules and feature selection [32]. The first arrow indicates the mapping of probability vectors to main vectors and the second arrow shows the elimination of some features which have zero variance. The boxes on the extreme right contain the derived rule. The first rule is a negation as it is derived from target values equal to zero.

For one target node, we consider all the possible nodes in the Boolean network (except the target) as the input nodes. Here, we have to find the causal elements of the target node and some strategy has to be adopted. Some nodes automatically get discarded if they are assigned a don't care value in the main vector. We can further analyze to see which other nodes can be eliminated, leaving only the true parents of a node in the corresponding Boolean rule. If a feature value has no variation in all the main vectors, associated with all the possible output values of node 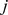, then we can discard that feature from a rule, as it does not contribute any discrimination power towards a rule. As an example, let's refer to Figure 3. Here, as the variance in feature 2 values is zero, it has no discriminatory power to decide between the two output values and hence, we can discard it when generating the final set of rules.

#### Rule Simplification

After the unnecessary literals/parent nodes are eliminated from a rule, we simplify the rule. As an example consider the rule (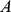 AND 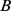) or 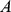. This rule is equivalent to 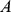. Hence, we compare every conjunct in the disjunctive normal form with every other conjunct and simplify the rule, wherever applicable.

#### Computing Training Accuracy Rate

After learning Bernoulli mixtures from input data for one node, each mixture is converted to its corresponding main vector. We learn mixtures for each value ‘0’ and ‘1’ of the target output values separately. It should be noted here that we generate a separate set of rules for both target labels. Ideally, the rules generated for the zero class should be a negation of the rules generated for the one class label. However, for real datasets it is not the case as we are generating the mixtures from positive and negative data points independently. Here, some strategy is required for selecting the best set of rules and it can be done by comparing a predicted rule's accuracy against the data comprising the training set. Suppose for one rule, 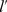 represents the total parents in a predicted rule. Ideally, the training set should have 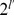, set of input combination values with corresponding output values. However, it is unlikely that all such data points are present in the training set. Also, many combinations are repeated several times. In this case, we compute the error rate of a rule from the training set by counting the fraction of incorrect predictions, for each unique combination of input data values, present in the training dataset. The expression is given by:
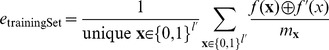
(14)Here, 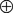 represents the XOR operation, 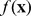 represents the actual output value (from data) corresponding to input 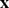, 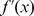 represents the output of the predicted rule for a given input 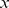 and 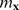 is the total number of training points whose value is 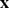. The rule with minimum training error is then chosen as the predicted rule.

#### Selecting 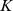, the Number of Mixtures to Use

As mentioned earlier, one of the parameters of our model is 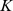, the total number of mixtures. We select the optimum value of 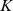 for a mixture model by starting with a very large value of 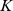 and letting the regularized version of EM, converge to an optimum number of mixtures. The regularization automatically eliminates some mixtures from the model. The best value of 
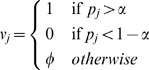
 is the one for which a rule has a minimum training set error (as given by 14).

**Figure 4:**
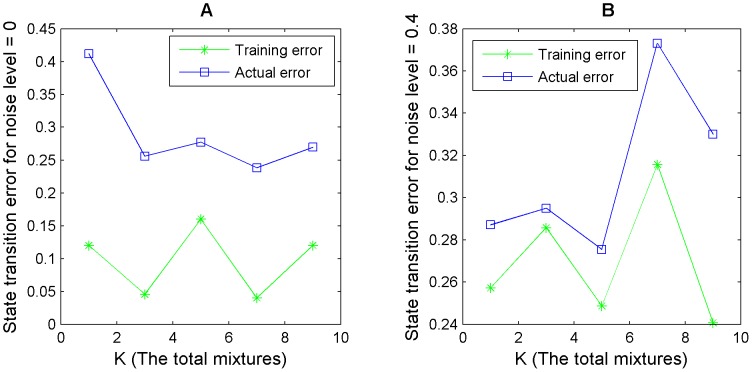
Model selection strategy adopted for choosing the best value of 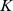 using the training set error as a criterion for selection. This figure is a plot of values shown in Table 2. Here the training error along with the state transition error for different 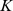 values is plotted for noise levels 0 and 0.4. The rules were generated for node 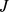 (indegree 9) of the 10 node network. We can see that generally, a higher training set error corresponds to a higher state transition error and vice versa.

For a single value of 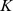 we might end up with less than 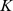 clusters as the regularized EM algorithm looks for mixtures with an optimum cross entropy value and pushes the individual probabilities in a mixture towards 1 and 0. If we repeat the experiments with different 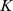 values, then each value might generate a different rule from data. In this case we have found that the training set error is a suitable measure for selecting the best rule. In 14, instead of simply counting the number of misclassified points by a rule, we count the number of unique points that are misclassified and weight them according to their proportion in data. This gives us a measure for generally assessing how good a rule fits the data. In case of very noisy data, this measure can lead to over-fitting of data, as the rule will also start fitting the noise and spurious observations. However, the regularized version of EM should be able to counter this by looking for probability vectors that are closer to the corners of the hypercube, rather than at the edges, i.e., more towards 0 and 1 rather than towards 0.5. Later in the results section, we illustrate different values of training set error for a given rule and the corresponding actual rule error for different 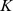 values (see Table 2 and Figure 4). The observations show that the training set error can be used as a good indicator of how well a rule explains the data and hence it can be taken as a criterion for model selection.

**Table 2:** Comparison of training error vs. actual error of a rule in model selection for different 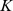 values.

	noise = 0		noise = 0.1		noise = 0.3		noise = 0.4	
	selected 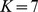		selected 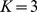		selected 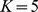		selected 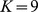	
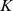	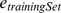	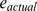	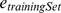	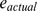	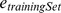	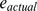	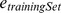	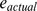
1	0.12	0.41	0.17	0.28	0.36	0.51	0.26	0.29
3	0.05	0.26	**0.03**	**0.22**	0.34	0.62	0.29	0.29
5	0.16	0.28	**0.03**	0.25	**0.23**	**0.33**	0.25	**0.28**
7	**0.04**	**0.24**	**0.03**	**0.22**	**0.23**	**0.33**	0.32	0.37
9	0.12	0.27	0.07	0.25	0.25	0.29	**0.24**	0.33

This table indicates the error of a rule as obtained from the training data and the corresponding actual state transition error for different noise levels and different values of 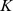, where 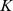 is the total mixtures in a Bernoulli mixture model. If lower training error also corresponds to lower actual rule error then training error can be taken as a good measure to assess the error of a rule and hence it can be used as a criterion for selecting 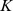. However, low training error corresponding to high rule error indicates over fitting. The results are shown for the 10 nodes network, for node 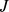 with indegree 9. The value of 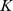 for which the training error is minimum is chosen as the final value of this parameter. The lowest error is highlighted for each column. The strategy works quite well for low noise levels and reasonably well for higher noise levels. The plot for noise levels 0 and 0.4 is also illustrated in Figure 4.

It should be noted here that this problem is a little different from a a typical machine learning problem, where we divide the data into training, validation and test sets. In our current scenario, we are required to output a rule for a target node when given the input data. The actual target rule is not used anywhere during the rule generation phase.

#### Complexity of ReBMM

Here is an analysis of the complexity of ReBMM algorithm.

- EM algorithm for one rule: 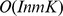

Here, we are assuming that we have 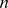 nodes in the network and 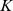 Bernoulli mixtures, generated from 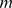 data points. 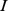 is the total iterations of EM algorithm.

- Selection of parent nodes: 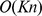

- Rule simplification: 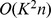

The 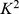 term is involved as every conjunct in a rule is compared with every other conjunct in the rule.

- Training set accuracy rate: 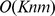.
- Generating a single rule: 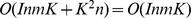

as generally 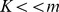. The above expression is the sum of the first 4 items.

- Reverse engineering an entire network of 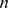 nodes: 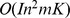

- Overall complexity: 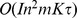

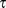
 is the number of times the simulations are repeated for model selection (for parameter 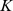).

From this analysis we can see that time complexity of ReBMM is quadratic in the total number of nodes of the network and linear in the total number of input data points. Generally, we run EM for 200 iterations and normally the value of 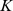 is also very small, i.e., less than 20. The efficiency of the algorithm is, hence, mainly affected by the total number of data points and the total number of nodes in the network.

### Evaluation of the Generated Boolean Network

In order to evaluate the performance of a method that reverse engineers a Boolean network, an assessment criterion is required. Researchers have suggested various measures, some based on the correct detection of parents and some based on the correct generation of truth tables. Popular measures include precision, recall and state transition error. All of these error measures have their strengths and weaknesses. We used all the three measures to evaluate our generated network and we discuss them next. Here, we are assuming that 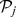 is the set of parents/variables involved in the actual rule for node 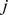, and 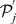 are the parents found by the reverse engineering method. Also, 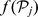 represents the actual rule governing the behavior of node 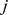 and 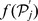 is the predicted rule against node 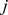.

#### State Transition Error

The state transition error 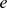 is computed by generating truth tables of both the actual rule and the predicted rule for a node in the network and then averaging it over all nodes in the network. In the challenge launched on causality, regarding the correct detection of a Boolean network, the challenge organizers suggested the same error measure for assessing different reverse engineering techniques. Formally, we can define the state transition error for the 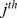 node, whose actual Boolean rule is given by 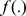 and the predicted rule is 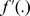, as:
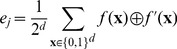
(15)Here, 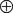 denotes the XOR operator. Also, 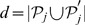, the total number of variables involved in both rules. At a first glance, the state transition errors seems like an appropriate choice for measuring the quality of an inferred network, e.g., consider two rules:

Actual rule 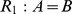


Predicted rule 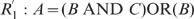


The above two rules are equivalent, even though the predicted rule involves a second variable 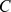. 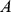 has the same truth tables for both cases, leading to zero state transition error. However, consider the following example:

Actual rule 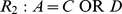


Predicted rule 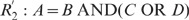


If 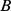 is a constant in the network, whose value is set to 1, then the two rules are equivalent. However, the state transition error is 5/8.

#### Precision and Recall

Precision 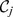 and Recall 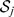 for the 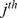 node are defined as:
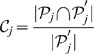
(16)

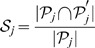
(17)Informally, precision computes the fraction of variables in an inferred rule, which are also present in the original rule and recall (also called sensitivity) measures the fraction of parents of node 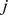 that are correctly detected by an algorithm. We can calculate the average of precision and recall for all the nodes to compute an overall accuracy measure for an inferred network. The two error measures are appropriate for evaluating the network structure identified by an algorithm. The limitation associated with these accuracy measures can be seen from the two rules 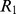 and 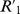. Here, the two rules are equivalent, however, precision is 1/2 and recall is 1.

## Results

This section presents the results of simulation of ReBMM on synthetic as well as real life datasets. The generated networks were evaluated using state transition error, precision and recall measures. The software used for these simulations was implemented using Matlab and is available upon request. This software generates the rules for individual nodes in the network and its state transition error is computed using the code provided by the organizer of causality challenge, Isabelle Guyon [23]. The code is available at http://www.causality.inf.ethz.ch/pot-luck.php. We also modified this code to compute precision and recall for the networks.

### Simulated network of 3 and 5 nodes

**Figure 5:**
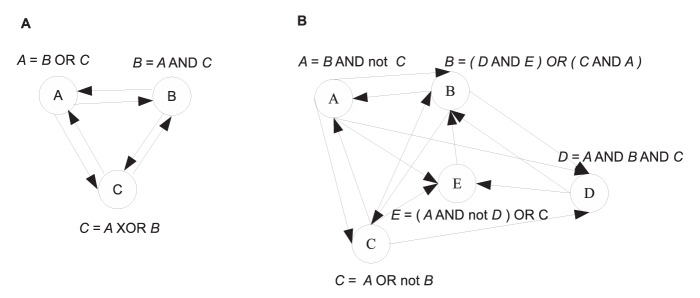
Network of 3 and 5 nodes.

In order to evaluate the performance of ReBMM we constructed a very simple network of three nodes (see Figure 5) and generated synthetic data synchronously from this network by choosing 3 initial unique states. Even though this network is extremely simple, it covers 3 possible logical relations involving logical AND, logical OR and XOR relationships. ReBMM allows perfect reconstruction of this network, which shows that it can extract linear (AND, OR) as well as non-linear relations (XOR).

After success with a 3 node network, we increased the number of nodes in the network to 5. The network is shown in Figure 5. Here, node 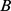 has an indegree of 4 and nodes 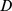 and 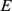 have indegrees of 3. For this case also, ReBMM correctly identified the given network with 0% error rate. All rules were correctly identified, leading to zero state transition error and precision and recall of the network being 100%.

### Network with 10 nodes and varying indegrees

**Figure 6:**
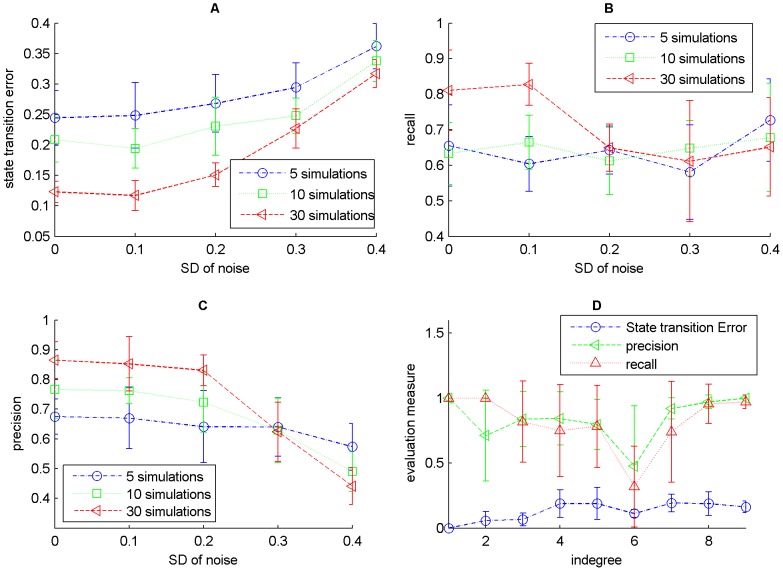
Evaluation of the network with varying indegrees using ReBMM. Figure A is the noise level vs. state transition error, Figure B is a plot of noise level vs. recall and Figure C indicates the noise level vs. precision. Noise level is the standard deviation 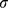 of Gaussian distribution with zero mean. In Figure D different evaluation measures are plotted for different indegrees for 30 simulations and noise level 0.1.

The effectiveness of ReBMM can be assessed by evaluating its performance by varying the indegrees of different nodes in the actual Boolean network. We took the network of nodes, generated from the logical rules shown in Table 2. Here we have different rules for each node and the indegree, of the nodes in the network, varies from 1 to 9. From this network we generated time series data by randomly taking different initial points. Experiments were repeated for 5,10 and 30 simulations, each simulation having 15 time steps each, allowing the network to reach a stable cycle. In order to simulate real life data we also added different noise levels to this data by generating random values from a Gaussian distribution with mean zero and varying standard deviations and then thresholding to the nearest 0 or 1 value. The different evaluation measures for different number of simulations are shown in Figure 6. The displayed results are an average over 10 runs.

**Table 3:** Network with 10 nodes, having varying indegrees.

RULES
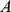 = 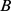
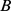 = not 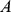
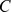 = 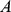 OR not 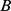
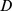 = ( 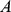 AND not 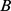 ) OR ( 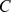 )
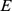 = ( 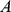 AND 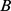 ) OR (not 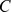 AND 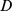 )
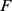 = ( 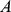 AND not 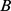 ) OR ( 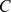 AND not 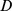 OR 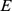 )
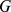 = ( 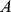 AND not 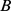 ) OR ( 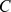 AND not 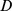 OR not 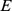 ) OR 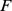
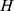 = (not 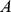 OR not 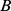 ) AND (not 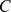 AND 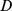 AND not 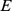 ) OR ( 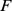 AND 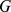 )
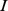 = (not 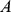 OR not 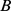 OR 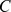 ) AND (not 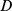 AND not 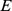 ) OR (F AND G OR H)
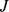 = (not 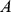 OR not 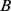 OR 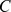 OR 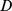 ) AND ( 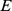 AND not 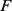 ) OR ( 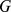 AND 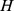 OR 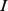 )

We used this network to generate data with different noise levels and reconstructed a Boolean network from this synthesized data. Precision, recall and state transition error for the generated network were measured using this network as the reference system.

We can see that for low noise levels, as the number of simulations is increased, the state transition error decreases and the precision and recall increase, indicating more accurate detection of parent nodes. With increase in noise levels, the precision of 30 simulations deteriorates significantly as compared to 5 and 10 simulations. More noise means more inaccuracies in data, leading to poor performance. Figure 6 (panel D) shows the performance of individual nodes in the network for 30 simulations and noise level 0.1. For lower indegrees, the state transition error is low and as expected, it is a little higher for higher indegrees. It does not vary much for indegrees other than 6, for which it is quite low. On the other hand, precision and recall exhibit a different behavior. The precision and recall for indegree 6, i.e., node 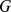, are quite low because of the nature of this node's corresponding rule. When the truth table for this rule is formed the output column has a majority of 86% ones. Depending upon the initial state of a simulation, our method predicts the rule ‘
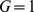
’ in many cases, leading to zero recall and zero precision. On the other hand the state transition error of this node is low, indicating that it fits the data quite well.

**Figure 7:**
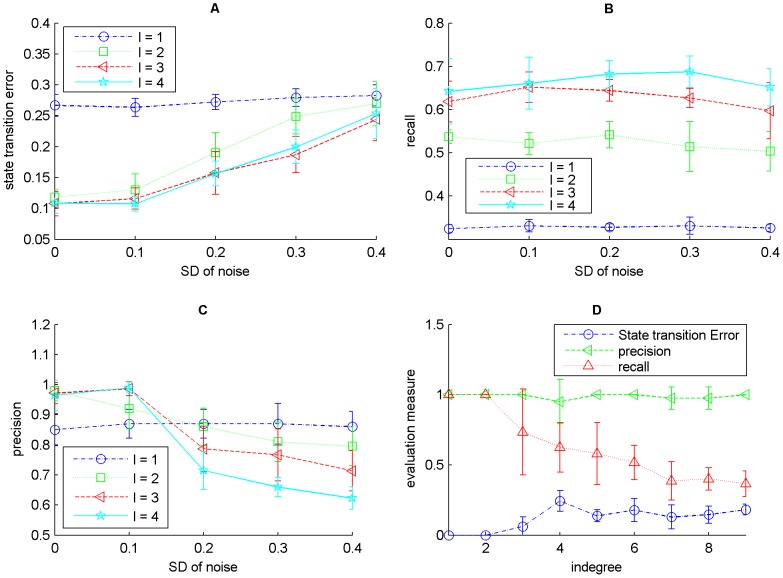
Evaluation of the network with varying indegrees using REVEAL. Figure A is the noise level vs. state transition error, Figure B is a plot of noise level vs. recall and Figure C indicates the noise level vs. precision. Noise level is the standard deviation 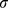 of Gaussian distribution with zero mean. In Figure D different evaluation measures are plotted for different indegrees for noise level 0.1 and indegree 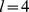. All experiments were carried out using 10 simulations.

**Figure 8:**
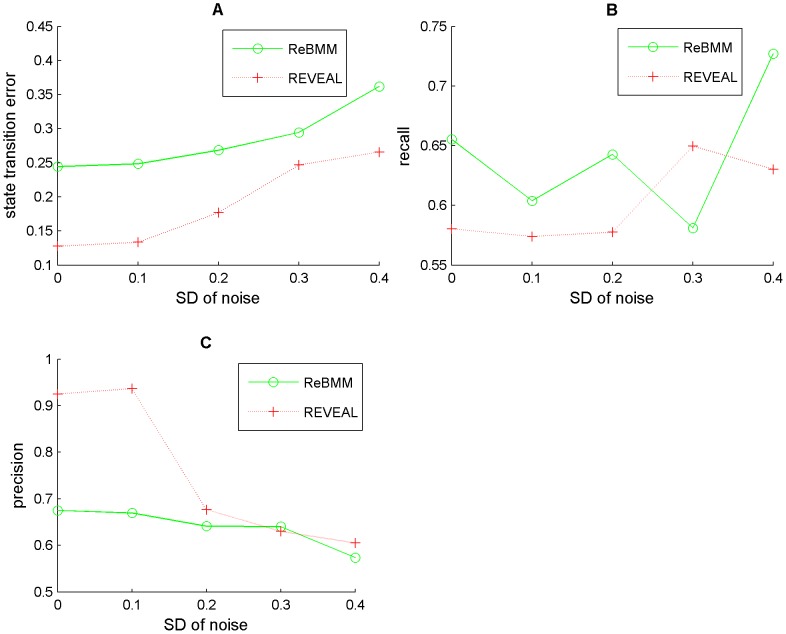
Comparison of REVEAL and ReBMM using different measures. Figure A compares noise vs. state transition error, Figure B is a plot of noise level vs. recall for the two methods and Figure C indicates the noise level vs. precision. Noise level is the standard deviation 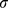 of Gaussian distribution with zero mean. The total simulations in all experiments is 5 and the indegree for REVEAL is 4.

Figure 7 shows the performance of REVEAL on the same network. REVEAL seems to exhibit the same type of behavior as ReBMM, with precision deteriorating significantly for 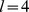, at high noise levels. Figure 8 compares REBMM and REVEAL (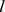 = 4) for different noise levels. We can see that the performance of REVEAL is significantly better in terms of state transition error, even for higher noise levels. However, the recall for ReBMM is higher for high noise levels and precision is almost the same. This shows that a method like REVEAL that conducts a brute force search over the space of all possible rules is a better choice when the data is clean and the indegrees of nodes in the network are small. However, an optimization technique like ReBMM performs better in case of noisy data in terms of accurate detection of parent nodes.

In Table 2, we illustrate our model selection strategy for choosing 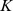 for the network of 10 nodes. As mentioned earlier, we need a scheme for selecting an optimum value of 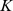, the number of mixtures to use, when generating a rule. We generate rules for different 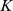 values and assess them using training set error as given by 14. The value of 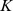 for which the training set error is minimum is chosen for rule extraction. In case two values of 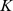 correspond to the same training error, we pick the smaller of the two. Table 2 shows the training set error of different rules, along with their corresponding actual state transition error (given by 15) for various noise levels and different 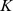 values. The experiments of this table were performed on the node with the highest indegree, i.e., node 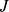 with indegree 9. The best error value in a column has also been highlighted. We can see that generally, a low value of training set error is a good indicator of a more accurate rule for clean data (noise = 0) and also when some noise is present in the data (noise 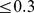). For a higher distortion of data (noise 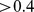), a rule starts to overfit the data but this measure still works reasonably well. The results of Table 2 are also plotted in Figure 4 for noise levels 0 and 0.4. The plot clearly indicates a positive correlation between training set error and actual error for different 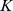 values, confirming that our model selection strategy is sound.

### Simulations for SIGNET Dataset

The SIGNET dataset was launched in conjunction with the causality challenge [21], [22] and is part of the workbench's dataset. The data describes interactions between 43 Boolean variables in a plant signaling network. It consists of pseudo dynamic simulations of 43 Boolean rules. The initial vector for each simulation was generated randomly and the rest of the vectors in the time series were derived using an asynchronous update scheme. Each simulation is represented by a 10
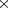
43 matrix. The goal is to recover the original set of rules, which generated this dataset.

Table 4 shows the results on the SIGNET dataset for different number of simulations. As the number of simulations increases, the state transition error decreases and the precision and recall increase. Again this result is expected as our underlying probabilistic model becomes more and more accurate, with more incoming data.

**Table 4:** Results for different number of simulations of SIGNET network.

No.	Simulations	Error	Recall	Precision
1.	50	.06	.79	.83
2.	100	.03	.90	.95
3.	300	.02	.93	.93
4.	500	.01	.94	.92

The table shows the state transition error, precision and recall for the generated network using ReBMM. We can see that increasing the number of simulations leads to a more accurate reconstruction of the network. The state transition error reduces to only 1% for 500 simulations and precision and recall are above 90%.

### Comparison of Results

In order to compare the performance of ReBMM with other methods, we ran our simulations on SIGNET dataset using two other algorithms, i.e., REVEAL and brute force search method as proposed by Akutsu *et al.* [30]. The results are summarized in Table 5. All experiments were carried out on a DELL 520 laptop with intel core 2 duo 1.66 Ghz processor, and 1.5 GB RAM. We can see that the state transition error is below 0.02 and precision and recall are above 90% for all methods, however, there is a big difference in time to accomplish this. For REVEAL, we restricted the parameter, 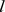, the number of parents of each node to 3. It should be noted here that 58% nodes in this network have an indegree of 1. REVEAL easily infers the rules correctly as we have restricted the indegree to a maximum of 3. However, for nodes with larger actual indegrees, REVEAL will not be able to generate the rules correctly. The last row of Table 5 illustrates the result of running Akutsu *et al.*'s brute force search to determine Boolean rules, governing an underlying Boolean network. We searched for rules connected together by simple gates ‘AND’, ‘OR’ and ‘NOT’, and again restricted the indegree of all nodes to a maximum of 3. In case of Akutsu's brute force search, it takes almost one minute to run it for l = 2. However, increasing the value of l to 3, increases the time to around 1.5 hrs, as the complexity of this method also involves a factor of 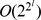. We cannot run this algorithm for network of nodes with higher indegrees in real time. On the other hand, in case of ReBMM, the method has no requirement for the assumption of indegree of a node, and hence can work for networks with higher connections also.

**Table 5:** Results for different methods on the SIGNET dataset.

Method	Error	Recall	Precision	Time
ReBMM	0.0210	0.9283	0.9283	1 hr, 16 m
REVEAL	0.0233	0.9442	0.9380	2 hr, 41 m
Search	0.0174	0.9132	0.9767	1 hr, 36 m

We ran three different methods, i.e., ReBMM, REVEAL and Akutsu's search method to reconstruct the SIGNET network. We can see that ReBMM takes the least amount of running time as compared to other methods. The state transition error is comparable for all three methods, however, precision is the highest for the search method.

### Reconstruction of a Boolean Network from Yeast Cell Microarray Dataset

**Figure 9:**
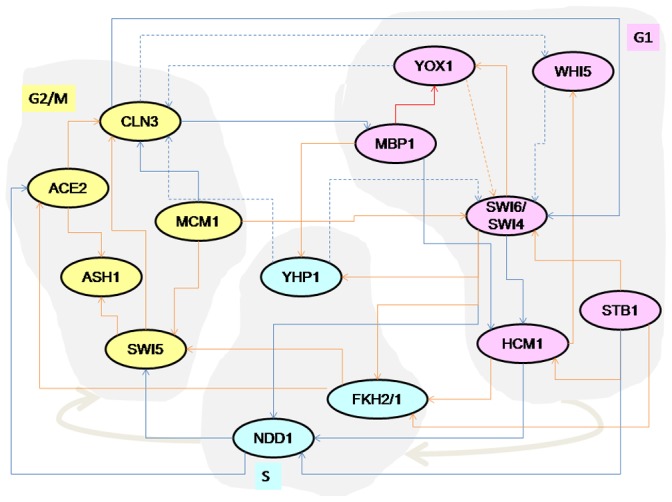
Transcription factor network of the yeast cell cycle inferred by Orlando *et al.* and Pramila *et al.* Solid lines indicate activating interactions and dashed lines indicate inhibitory interactions. The transcription factors of the cell cycle time line are indicated by the gray blobs (G1
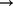
S
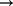
G2/M). The interactions shown in red were correctly identified by ReBMM and the interactions shown in blue were not identified.

Our last set of experiments were carried out on the cell cycle transcriptional network of budding yeast as suggested by Orlando *et al.* [31]. We reconstructed a Boolean network from the microarray data from alpha, cdc15 and cdc28 experiments conducted by Spellman *et al.* and Cho *et al.* [32], [33]. The data was binarized using the same method as Muacher *et al.*, via our own implementation of k-means algorithm, with the total centers being set to 2 [17]. Similar to their experiments we also added 16 randomly selected genes, along with the 16 actual genes of the biological network and ran ReBMM to determine the interactions of the Boolean network. We also repeated the experiments 25 times to assess our method, every time taking a different set of randomly selected genes. To compute precision and recall we used as reference, the published network of 16 genes as given by Orlando *et al.* and Pramila *et al.* [31], [34] and it is shown in Figure 9.

**Figure 10:**
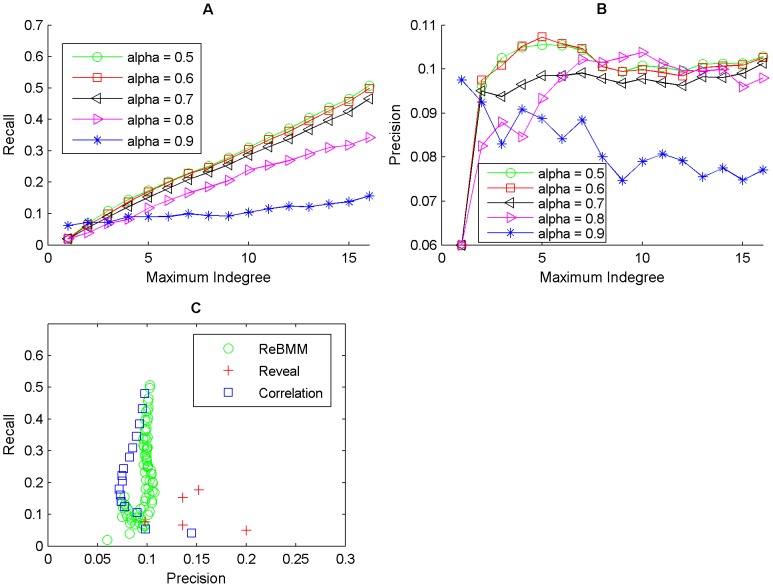
Reconstruction of network for the cdc15 experiments of the yeast cell cycle microarray data. Figure A shows the recall against maximum indegree for ReBMM for different threshold values, 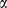. Figure B illustrates the plot for precision. The comparison of ReBMM, correlation and REVEAL is displayed in Figure C. Clearly the performance of ReBMM is better than the other two methods in terms of precision. REVEAL has the highest precision but the recall is very low.

**Figure 11:**
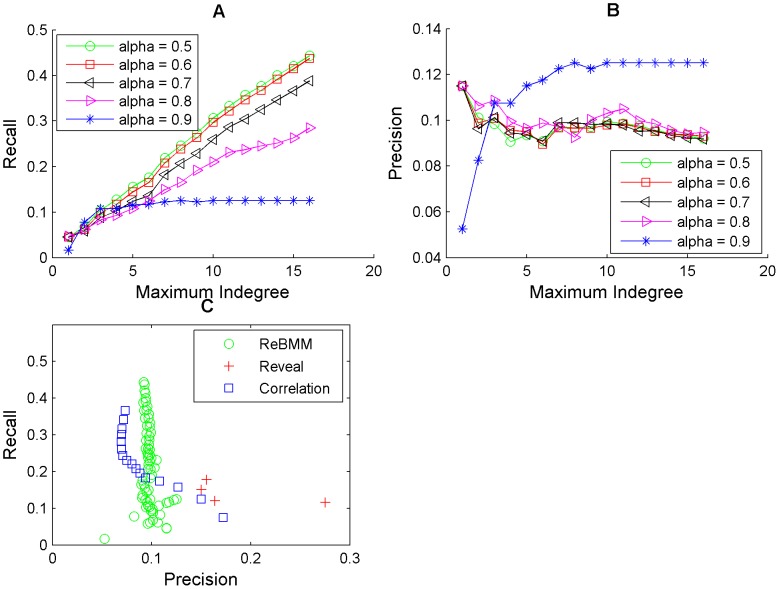
Reconstruction of network for the alpha experiments of the yeast cell cycle microarray data. Figure A shows the recall against maximum indegree for ReBMM for different 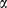 values. Figure B illustrates the plot for precision. Figure C illustrates the comparison of ReBMM, correlation and REVEAL. Clearly the performance of ReBMM is better than the other two methods in terms of recall.

**Figure 12:**
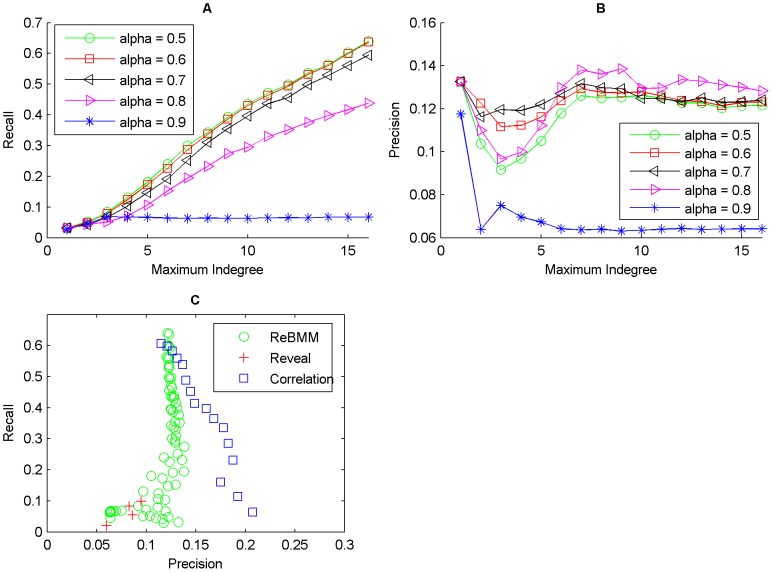
Reconstruction of network for the cdc28 experiments of the yeast cell cycle microarray data. Figure A shows the recall against maximum indegree for ReBMM for different 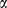 values. Figure B illustrates the plot for precision. The comparison of ReBMM, correlation and REVEAL is displayed in Figure C. Here the performance of correlation is better than the rest of the methods.

Figures 10, 11 and 12 show the average accuracy of the results of reconstructing the network by using different values of the threshold 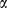, over 25 different runs of the experiment. We restricted the indegree of each node to a maximum value and repeated the experiments for different values of 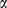. For all the simulations, the reconstructed network has low precision but reasonably good recall. This observation is similar to that of Maucher *et al.*, who also report low precision values on the same network using correlation. For real life data, values of 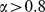 reconstruct a network with low recall and precision and 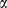 set to 0.5 or 0.6 give better results. As the indegree increases, the recall also increases. The precision on the other hand does not vary much.

To compare ReBMM's performance, we also reconstructed the same network using REVEAL and the correlation method. Figure C of Figures 10, 11 and 12 shows the performance of REVEAL, correlation method and ReBMM. The indegree of REVEAL was varied from 1 to 6. We can see that all methods,i.e., ReBMM, correlation and REVEAL have higher recall as compared to precision. This is of course under the assumption that our reference network represents the true biological situation. Interestingly, all three methods point to the presence of more network connections, as compared to the true network. The comparsion results show that the overall performance of ReBMM is better than correlation measure for alpha and cdc15 experiments. For cdc28 experiments, correlation method outperforms the other two methods. Clearly, REVEAL has higher precision as compared to both correlation and ReBMM but the recall is very low. However, we found it unpractical to run REVEAL for indegrees higher than 6.

In Figure 9, we have drawn the interactions detected from one sample run of ReBMM with alpha set to 0.6. Here we can see that some of the dependencies, e.g., YOX1, FKH1, e.t.c. are correctly identified. However, the parents for MBP1 and MCM1 could not be detected correctly. ReBMM also infers interactions that are not actually present in the reference network. Here our assumption is that the reference network reflects the true biological situation, which may not necessarily be true.

### Comparison of Running Time

**Figure 13:**
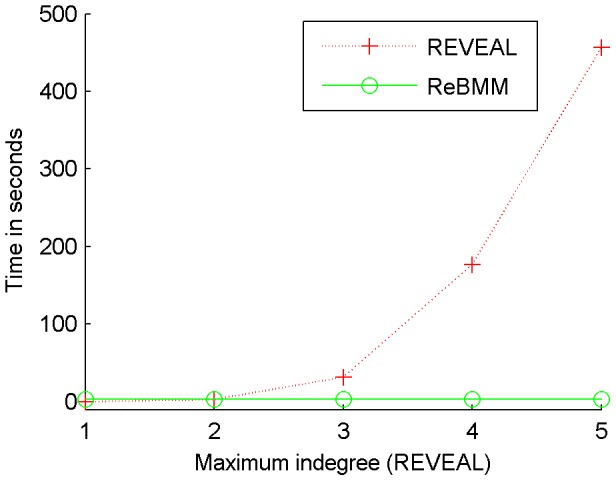
Comparison of running time of REVEAL and ReBMM. ReBMM's running time is independent of the maximum indegree of nodes in the network. REVEAL's running time increases markedly with the increase in the assumed indegree.

Figure 13 shows a comparison of running times of REVEAL and ReBMM for a network of 32 nodes, where the rules for 16 genes were determined. The time taken for ReBMM is independent of the assumed indegree of a node. On the other hand REVEAL has to make 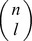 subsets to determine the cross entropies between variables. Similarly, all possible rule combinations have to be tried out in order to determine the correct rule that fits the given data. Hence, the running time of REVEAL increases significantly with the increase in 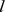. It should be noted here that we have not stressed on optimizing the written code for the implementation of the two methods. The time graph, hence, does not depict the best running times of an efficient implementation of the reconstruction algorithms. It is only intended to illustrate that there is a marked increase in running time of REVEAL with the increase in the assumed indegree of nodes in the network.

## Discussion and Conclusions

In this paper we have introduced a novel, simple and intuitive technique for reverse engineering a Boolean network from a probabilistic model comprising Bernoulli mixtures. The salient features of our method are:

1. We do not have to make an assumption about the number of parents of a node, and hence the complexity of ReBMM is not affected by varying the indegree of a node.
2. Changing the parameter 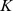, the total number of mixtures, does not reflect upon the number of parent nodes but determines the number of conjuncts in a rule.
3. Unlike other methods, we do not have to try all combination of Boolean functions to determine the logical rule. Here, the main vector, derived from a Bernoulli mixture, determines the Boolean rule in a natural and intuitive fashion.
4. The accuracy of ReBMM is comparable to that of other conventional methods
5. The complexity of many conventional reverse engineering techniques has an exponential term or a high degree polynomial, involving the assumed number of parents, whereas ReBMM involves a quadratic term, of the total number of nodes in the network.

The performance of ReBMM was evaluated using synthetic datasets generated from an artificial set of rules and an actual plant signaling network. In another set of experiments, real microarray data from the yeast cell cycle experiments was also used. We found that ReBMM is efficient and gives comparable results to other traditional approaches like REVEAL and a later approach like the correlation based method. ReBMM can easily reconstruct Boolean networks with indegrees higher than 5. On the other hand, REVEAL conducts a brute force search of all possible input node combinations and all possible functions, which makes it impractical to be used in scenarios where the network size is large and the indegree of a node is high. Apart from the computational complexity, a major problem with REVEAL for higher indegree nodes is that a large amount of data is required to estimate the cross entropies between pairs of variables. With limited data, these entropies cannot be computed reliably and can lead to the incorrect detection of dependencies between the various nodes in the network. In light of our experience we recommend using REVEAL for only very small networks with limited indegrees and where the data is clean and noise free. On the other hand ReBMM can be used when we have larger networks with higher indegrees.

In case of real life microarray data, the existing methods such as ReBMM, correlation and REVEAL can act as a supplementary or complementary methods to microarray analysis. These methods may not have very high precision and recall values but still have the potential to pin point some of the important gene interactions from the data. It has also been pointed out by Lähdesmäki *et al.* that if there are two genes, 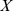 and 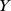 with similar expression profiles and if gene 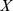 has peaks and troughs just before that of gene 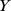, then all the afore mentioned methods would conclude that gene 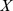 controls gene 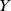, which might not be true in the real scenario. In such cases we need further data such as location data and functional studies.

In this work we also found that the correlation method of Maucher *et al.* is an efficient and effective method for computing dependencies between nodes in the network. However, it is not possible to find the exact rule governing a network using this method. Also, the correlation method is restricted to detecting parents of node whose rules are linear monotone functions. A method like ReBMM can be used in conjunction with this method to derive a complete Boolean network model.

As an extension to our work, we are looking at combining ReBMM with correlation and entropy based methods for the detection of parent nodes in the network and using Bernoulli mixtures for determining the rules governing the target node. For real experimental data like the yeast cell cycle microarray data, there is still need for developing more robust mathematical measures that can reliably detect the parent nodes when only a limited number of observations of the time series are available. Even though, Boolean networks are highly simplified models, they can still capture many important details of an actual biological network. We are also looking at extending this model to probabilistic Boolean networks. ReBMM's framework can lend itself naturally to modeling probabilistic Boolean networks using Bernoulli mixtures.
